# Common sequence variants affect molecular function more than rare variants?

**DOI:** 10.1038/s41598-017-01054-2

**Published:** 2017-05-09

**Authors:** Yannick Mahlich, Jonas Reeb, Maximilian Hecht, Maria Schelling, Tjaart Andries Petrus De Beer, Yana Bromberg, Burkhard Rost

**Affiliations:** 1 Computational Biology & Bioinformatics - i12, Informatics, Technical University of Munich (TUM), Boltzmannstrasse 3, 85748 Garching/Munich, Germany; 2 Department of Biochemistry and Microbiology, Rutgers University, 76 Lipman Dr, New Brunswick, NJ 08901 USA; 3 TUM Graduate School, Center of Doctoral Studies in Informatics and its Applications (CeDoSIA), Technische Universität München, 85748 Garching/Munich, Germany; 4 Institute of Advanced Study (TUM-IAS), Lichtenbergstr. 2a, 85748 Garching/Munich, Germany; 5 European Molecular Biology Laboratories, European Bioinformatics Institute (EMBL-EBI), Welcome Trust Genomes Campus, Cambridge, Cambridgeshire UK; 6 Institute for Food and Plant Sciences WZW–Weihenstephan, Alte Akademie 8, Freising, Germany

## Abstract

Any two unrelated individuals differ by about 10,000 single amino acid variants (SAVs). Do these impact molecular function? Experimental answers cannot answer comprehensively, while state-of-the-art prediction methods can. We predicted the functional impacts of SAVs within human and for variants between human and other species. Several surprising results stood out. Firstly, four methods (CADD, PolyPhen-2, SIFT, and SNAP2) agreed within 10 percentage points on the percentage of rare SAVs predicted with effect. However, they differed substantially for the common SAVs: SNAP2 predicted, on average, more effect for common than for rare SAVs. Given the large ExAC data sets sampling 60,706 individuals, the differences were extremely significant (p-value < 2.2e-16). We provided evidence that SNAP2 might be closer to reality for common SAVs than the other methods, due to its different focus in development. Secondly, we predicted significantly higher fractions of SAVs with effect between healthy individuals than between species; the difference increased for more distantly related species. The same trends were maintained for subsets of only housekeeping proteins and when moving from exomes of 1,000 to 60,000 individuals. SAVs frozen at speciation might maintain protein function, while many variants within a species might bring about crucial changes, for better or worse.

## Introduction

Single nucleotide variants (SNVs) constitute the most frequent form of human genetic variation^1^. Here, we focus on non-synonymous SNVs, *i.e*. genomic variants that result in single amino acid variants (SAVs) in protein sequences. Children differ by about two SAVs from their parents (*de novo* variation), while any two unrelated individuals can differ by as many as 10–20 K^2^. The vast majority (99%) of the known unique SAVs are rare, *i.e*. observed in less than 1% of the population^1, 3^. Only about 0.5% of the unique SAVs are common, i.e. observed in over 5% of the population^1, 3^. SAVs can impact protein function in many ways.

We might be inclined to classify SAVs according to what they affect or do not affect. Effects are commonly distinguished upon protein function and structure. This distinction has limited value because what changes structure often tends to affect function. Similarly, we might distinguish between the effect upon molecular function (*e.g*. binding stronger or not binding), upon the role of a protein in a process (native process hampered, blocked, or non-native role acquired), or upon the localization of a protein (*e.g*. protein makes it to the membrane or not). Again the problem of this distinction is that these aspects are coupled: for instance, effects upon molecular function and localization might affect the process or not. All of the above, we might classify as effects upon the protein. Unfortunately, from all experiments monitoring SAV effects in many model organisms, just a few tens of thousands effects are available in public databases. For a tiny subset of these, enough detail is available to consider all effect types (structure vs. function, molecular vs. process vs. localization). We might consider the effect upon protein as *molecular* as opposed to the effect upon the organism, such as diseases. Toward this end, the distinction is often made between SAVs that cause severe monogenic diseases^4^ (referred to as *OMIM-type SAVs*) or contribute to complex diseases^5^ and low-effect SAVs, which are only cumulatively linked to our phenotypic individuality^6^. This latter distinction is the only one for which ample data is available. Methods predicting the effect of SAVs differ in many ways, including in what experimental data they use and how they use it. Typically, the methods inherit the bias of the data with respect to the type of effect considered.

Almost any SAV will have some effect under some condition. However, some SAVs clearly have stronger effects. A set of SAVs with stronger effect is likely to affect more aspects of function (molecular, process, localization) simultaneously. For example, OMIM-like SAVs are assumed to largely affect the biological process through strong effects upon structure and/or molecular function (or localization). Many of these SAVs also affect the organism as a whole manifesting as disease. Prediction methods add another level of complexity: some methods provide values reflecting the strength of a prediction that correlates with the reliability of the prediction (i.e. its accuracy) and the strength of the experimental effect. Assume we analyzed two subsets of effect predictions from a method: those with predictions stronger than threshold T1 and those stronger than T2, where T2 > T1. For methods for which the score is well-balanced, two statements are true: (1) setT2 is predicted at higher accuracy than setT1, and (2) setT2 has, on average, stronger effect than setT1. No matter what conditions or populations we analyze, results valid at T1 are also valid at T2, as long as the thresholds are chosen in a regime in which the prediction method works.

Analyzing the effect of all known variants experimentally remains unfeasible. Computational methods may incorrectly predict the effect for some SAVs, but they successfully capture trends for large sets of SAVs^7–11^. Furthermore, computational predictions are available for all variants and inherit only some of the bias from today’s experimental techniques. Both experimental and computational assays often fail to infer the impact of variation on the organism as a whole from individual SAV molecular effects.

The 1000 Genomes Project (*1KG*)^12, 13^ sequenced 1,092 individuals from 14 populations recording about 268,115 SAVs. In August 2016, the MacArthur lab at the Broad expanded on this collection by reporting 7,599,572 SAVs between 60,706 people^3^. How much of this variation impacts protein function? Only *in silico* tools can fully address this question. Here, we present a comprehensive and detailed analysis of the known human SAVs (1KG) and of SAVs that differentiate human proteins from their homologs in other species (hominids, primates, rodents, and fly).

## Results

### Many 60KE SAVs predicted with effect

**Figure 1:**
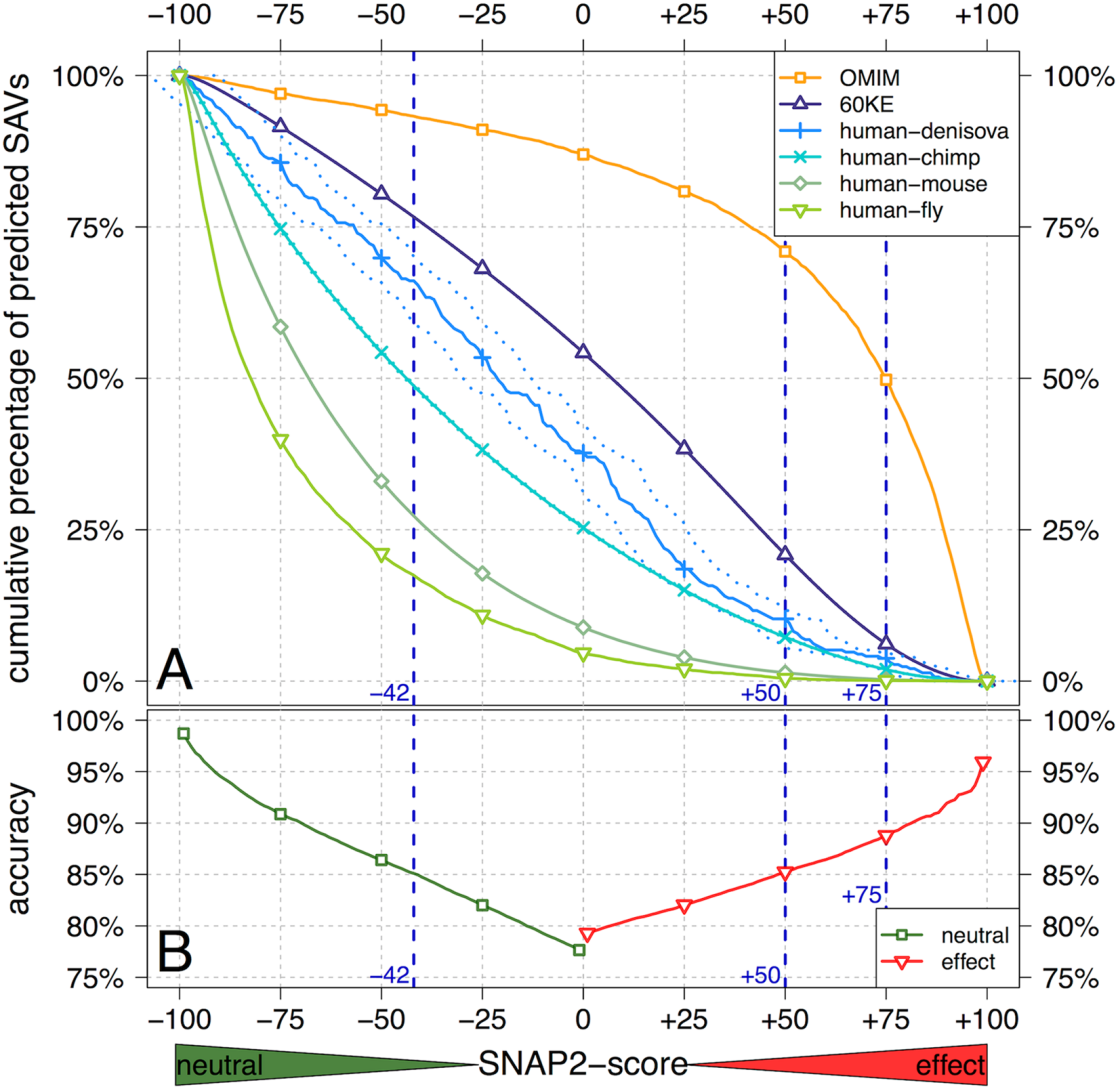
60KE SAVs predicted to have more effect than cross-species variants. SNAP2 predicts the effect of single amino acid sequence variants (SAVs) upon protein function: the higher the score, the more reliable the prediction (horizontal x-axis, toward right); the more negative, the stronger the prediction that the variant is neutral (horizontal x-axis, toward left). The top panel (A) gives cumulative percentages, i.e. the percentage of SAVs in a data set predicted above a certain value, *e.g*. for SNAP2-score ≥+75, about 6% of all 60KE SAVs are predicted to have an effect; at the same threshold about half of all disease-causing SAVs are predicted to affect function. For *60KE*, *denisova* and *chimp*, 99.7% confidence intervals (SNAP2-score ±3 standard error of mean) are indicated by dotted lines (indistinguishable for *60KE*, barely distinguishable for *chimp*, clearly visible for *denisova*). Lower panel (B) gives cumulative accuracy (red: effect-SAVs correctly predicted to have effects, green: neutral-SAVs correctly predicted); here the values accumulate from the extremes to 0*, i.e*. left-to-right for neutral (green −100 to 0) and right-to-left for effect (red +100 to 0); estimates from cross-validation using only molecular function^21^. For instance, at SNAP2-scores ≥+75 about 88% of all effect-SAVs are correctly predicted. On the other hand, variations between homologs in human and other species (human-denisova, human-chimp, human-mouse, and human-fly) were predicted to be much more neutral (all curves shifted toward lower left corner of neutral variants).

SNAP2 spreads its predictions into a wide interval of scores, in addition to predicting a reduced binary outcome (effect/neutral). The scale ranges from −100 (SAV strongly predicted as neutral) to +100 (SAV strongly predicted as effect–either deleterious or beneficial). For a binary prediction, SNAP2 is optimized for the threshold = 0 (*i.e*. neutral: −100 ≤ SNAP2-score ≤0 and effect: 0 < SNAP2-score ≤100). At this default threshold, 78% of the known neutral and 79% of the known effect SAVs are predicted correctly (Fig. 1 lower panel). Higher absolute values of scores imply more reliable predictions, as illustrated by the three example scores. For instance, zooming into more reliable neutral predictions at scores ≤−42 or into stronger effect predictions at scores ≥+50 raises the accuracy to 85%; going further to effect scores ≥+75 reaches 88% accuracy (Fig. 1 lower panel). We use these three examples throughout the manuscript to highlight trends. Note that to avoid two possible misunderstandings we point out that: Firstly, there is no single threshold for “strong” predictions, the higher the value the stronger the effect (below) and the higher the probability for the prediction to be correct. Secondly, zooming into some higher threshold such as SNAP2-score ≥75 does not imply that all with SNAP2-scores <75 are predicted neutral. Instead, we simply focus on a subset of strongly predicted SAVs.

We used the raw SNAP2-score to summarize our central results for all data sets cumulatively, *i.e*. showing at each SNAP2-score which percentage of the data set was predicted at or above that score. For instance, of the 7,599,572 60KE SAVs in healthy humans, for which predictions were available, 1,642,225 (about 21%) were predicted to have functional effects at a SNAP2-score ≥+50 (Fig. 1A, upper panel *60KE* purple line with triangle markers intersecting the blue vertical dashed line of score = +50). This threshold corresponds to an estimated accuracy of 85% (red line in Fig. 1B). On the other hand, SNAP2 predicted 100 − 77 = 23% of the 60KE SAVs as clearly neutral (Fig. 1A leftmost blue vertical dashed line of score −42) at an expected accuracy of 85% (Fig. 1B).

Another extreme point was SNAP2-score >+75 (Fig. 1, rightmost blue vertical dashed line): if we considered only SAVs predicted above this effect level, we would capture half of the OMIM SAVs (Fig. 1A, orange line with circular markers; Fig. 1B, 88% accuracy). For the same threshold about 1/15^th^ of all 60KE SAVs (496,854) were predicted to have an effect. Loosely put, one of every 15 SAVs in healthy individuals is predicted to have as strong an effect as the top 50% of known disease variants. Note that the OMIM SAVs were predicted by a version of SNAP2 that was not trained on any OMIM or HumVar SAVs (Methods). Another SNAP2 version, that did use such disease-related SAVs, predicted a much stronger effect (Supplementary Fig. S1).

SNAP2 has been evaluated in comprehensive cross-validation tests. However, the performance estimates provided here (Fig. 1B) depend crucially on what data is included in the assessment (they are higher when using OMIM-type SAVs and lower when using the small subset of experimental-only neutrals). The error estimates in performance curves for the 60KE data (Fig. 1A upward pointing triangles) were obtained by boot-strapping^14^, i.e. by testing how the results depend upon changes in the data set. Note that for all 60KE data these error estimates were visually indistinguishable from the curves shown (at a 99.7% confidence interval, i.e. SNAP2-score ±3 standard error of mean: *denisovan*: ±8.46, chimp: ±0.45, 60k all: ±0.06, 60KE common: ±0.80, 60KE rare: ±0.06).

**Figure 2:**
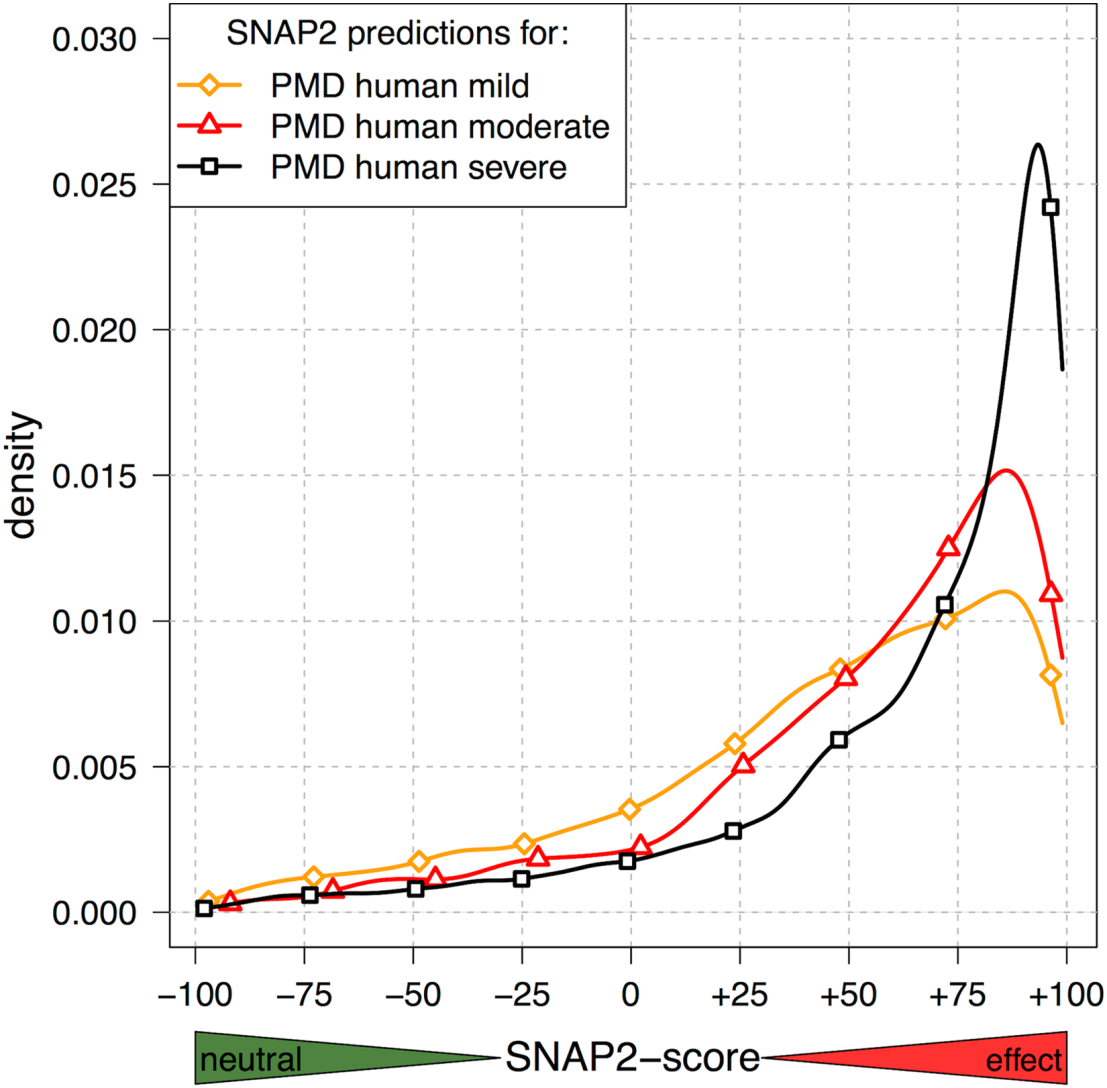
Higher SNAP2-scores imply stronger effect upon molecular function. We classified SAVs from the Protein Mutant Database (PMD) according to their impact upon molecular protein function into three classes (mild, moderate, and severe). Here, we repeat this analysis applying SNAP2 to the subset of human SAVs in PMD. We show density distributions, instead of cumulative. Although the three curves overlap, the shift is significant and consistent (black curve with most effect highest shift to right, orange curve with weakest shift most to the left). Thus, the SNAP2-score correlated with the strength of the effect upon molecular function.

For our previous method SNAP1, we have shown^6, 15^ that the SNAP-score correlates with effect strength, *e.g*. SAVs predicted closer to +100 tend to have stronger impact on molecular function than SAVs predicted closer to +50. Here, we confirmed the same for SNAP2 (Fig. 2). SNAP2 was trained on PMD variants, but never with fine-grained classification by degree of effect; instead SNAP2 was trained to classify binary labels (effect/neutral). Hence, the observed difference between mild, moderate, and severe could not have originated from SNAP2 training other than through the simple fact that stronger effects yield more consistent data and therefore effect strength is captured by the method. This correlation was exactly what we wanted to show.

### Cross-species variants predicted with less effect than variation within human

We analyzed all amino acid differences between human proteins and their *homologs* in other species (“cross-species variation”). For simplicity, we referred to those variants as to SAVs. There is an important caveat for the comparison between 60KE and cross-species SAVs. With the 60KE set we compared a population using essentially the same gene pool (pairs of people differ by few SAVs spread across their ~20 K proteins). In contrast, the cross-species comparison had to be limited to subsets of corresponding proteins. The size of this subset is inversely proportional to the evolutionary distance; *i.e*. fewer proteins for more distantly related organisms. To simplify: 60KE compared few SAVs in all proteins, while cross-species compared many SAVs in a few proteins.

We began with the extinct hominid *Denisova hominin*. In all orthologs, we considered the effect of substituting a *Denisova* amino acid that was introduced into the corresponding human protein back to the human reference amino acid. When using all available orthologs, the SNAP2 predictions for the human-denisovan cross-species SAVs were moved toward “less effect”, *i.e*. a lower fraction of SAVs was predicted with effect for human-denisovan than for the 60KE-set (Fig. 1A, human-denisovan below 60KE). This implied that sequence variants that became *fixed* in the modern human population were more neutral, on average, than the SAVs within the living human population (60KE). The strength of this shift depended on the SNAP2-score: the numeric difference was highest thresholds of 0 and the relative difference was highest around thresholds of +50 (Fig. 1A). The shift between 60KE and cross-species SAVs was increased with species divergence (Fig. 1A: curves shifted toward the lower left implying less effect for chimp, mouse, and fly). Note that any overlap with SNAP2 training data was excluded from this study (Supplementary Note).

**Figure 3:**
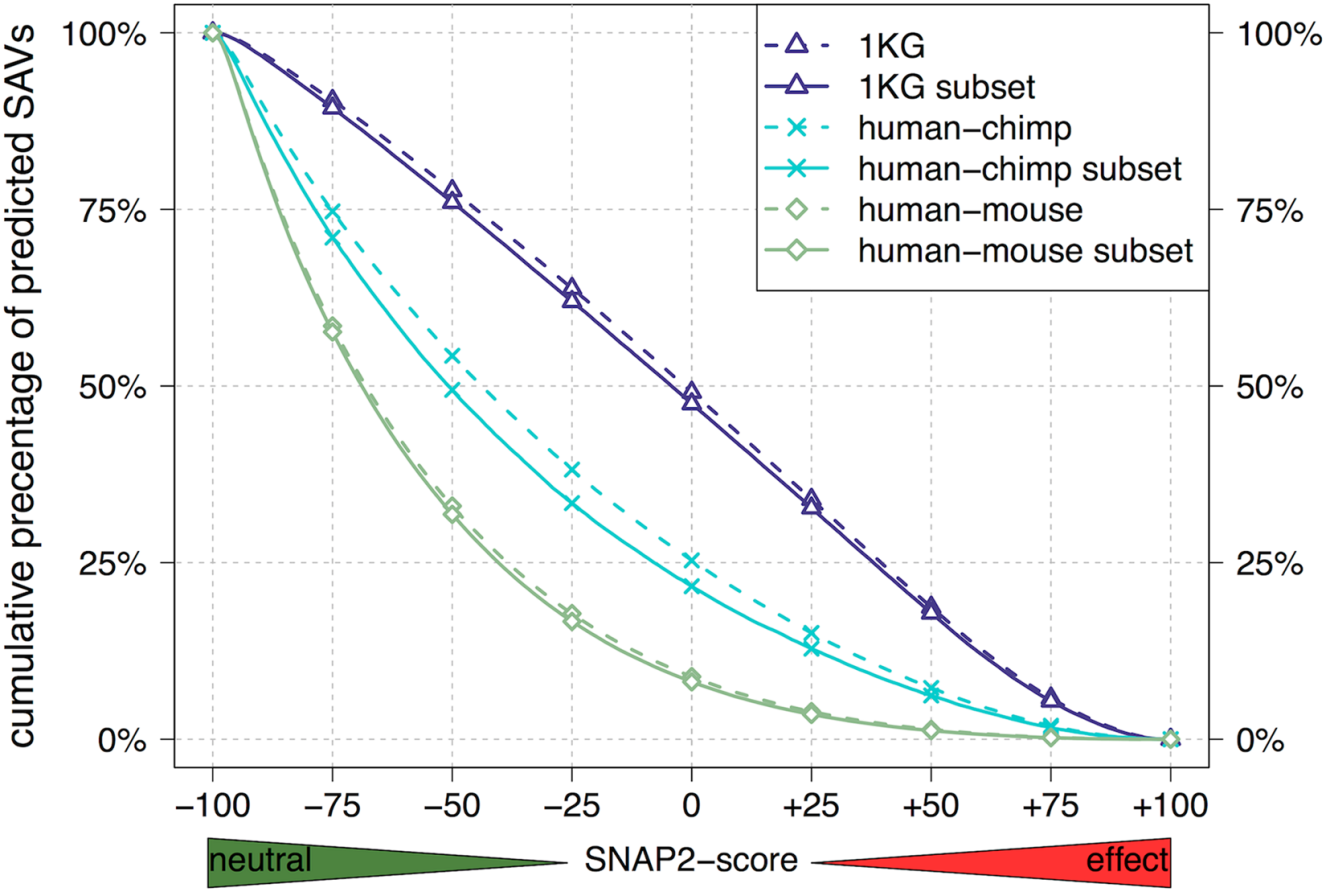
Subsets of “house-keeping” proteins confirmed findings for entire proteomes. We reduced the analysis to SAVs from subsets of orthologs between three organisms (human, chimp, mouse), and with SAVs observed in the 1KG data. For brevity, we referred to those as to “house-keeping” proteins. With respect to the observation for the entire data set (Fig. 1), the curves shifted less strongly, but the main trend remained: a higher fraction of the SAVs in cross-species comparison (human-chimp and human-mouse) was predicted as neutral than for the SAVs between healthy individuals (1KG). Furthermore, the shift between cross-species and 1KG was higher for larger evolutionary distances (more neutral for larger distance).

Next we addressed the problem of different “gene pools” (sets of proteins) for the comparison within human and between human and other species. Toward this end, we restricted our analysis to subsets of identical proteins, *i.e*. by restricting the SAVs to the subset of orthologs that were common to human, chimp, and mouse. The subset was restricted further by the constraint that 1KG SAVs be also observed in the same protein. The resulting curves were shifted toward “less effect” (curves higher in Fig. 1A than in Fig. 3); the standard errors of the mean increased only slightly (human: from 0.10 to 0.14; chimp: from 0.16 to 0.22; mouse: no change). However, the major characteristics of the curve shifts were not altered by the constraint to the subsets (Fig. 3: 1KG curve highest, higher evolutionary distance corresponds to lower curves). For less restrictive sets of orthologs, we observed similar trends (Supplementary Fig. S3).

### Common 60KE SAVs predicted with more effect than rare SAVs

Rare SAVs (allele frequency [LDAF] < 1%) dominate the set of unique SAVs in the 60KE data (7,530,337, *i.e*. 99.1% of all SAVs) and therefore dominate the overall analysis. If our objective were to assess the per-person effect instead of the per-SAV effect, we could have removed this bias by counting each SAV exactly once, *i.e*. by letting SAVs observed in ten individuals count ten-times more than those observed only once.

We might expect rare SAVs to sample the space of all possible SAVs. We investigated this through two sets of random SAVs introduced *in silico* into human proteins. One random set contained SAVs replacing the native amino acid by one of the 19 non-native amino acids. The other set was restricted to SNV-possible SAVs, *i.e*. amino acid substitutions that can be reached by a single nucleotide change (SNV; the set *SNV-possible* constitutes a subset of *19-non-native*). SNAP2 predicted the *19-non-native* SAVs to have slightly but significantly (two-sample Kolmogorov-Smirnov, KS, test: D = 0.068; n,n’ = 268,115; p-value < 2.2e-16) higher effects than SNV-possible SAVs (Supplementary Fig. S2).

**Figure 4:**
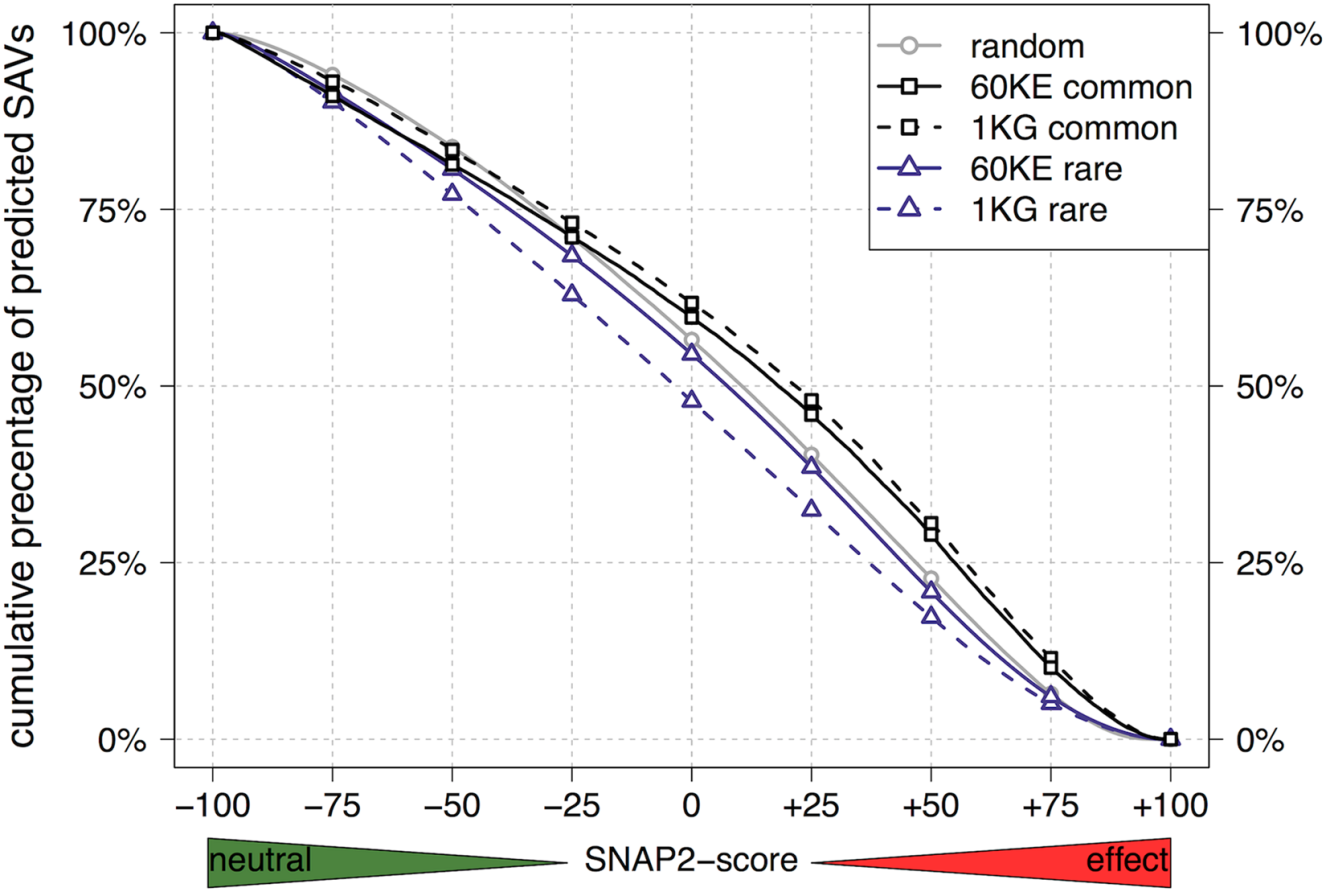
Common SAVs predicted with more effect than rare SAVs. We grouped SAVs by their observed frequency in 1KG and 60KE exome data: rare (LDAF < 1%: dark blue triangles), uncommon (1% ≤ LDAF < 5%: not displayed), and common (LDAF ≥ 5%: black squares). The potential mutational background for human was estimated by randomly selecting a set of SNV-possible SAVs (gray circles). The curves for rare SAVs were similar to the results for all SAVs (Fig. 1, purple triangles for 60KE) since counting only unique SAVs the results were dominated by rare SAVs. Rare SAVs were predicted below randomly chosen SNV-possible SAVs, although the recent 60KE set came close to random. In contrast, the set of common SAVs remained substantially above the random curve for both common-1KG and common-60KE (Kolmogorv-Smirnov, estimated p-value < 2.2e-16 in both cases).

One important aspect in the shift from the analysis of rare SAVs for 1000 people (1KG) to that for 60,000 people (60KE) was that the effect predicted for these variants by SNAP2 was very similar to the effect predicted for the random subset of SNV-possible SAVs (Fig. 4: solid blue line “60KE rare” much closer to gray random than dashed blue line “1KG rare”). The differences between the curves were small in absolute terms but statistically very significant (two-sample KS test, D = 0.033; n_*rare*_ = 7,530,277, n_*random*_ = 268,115; p-value < 2.2e-16).

**Figure 5:**
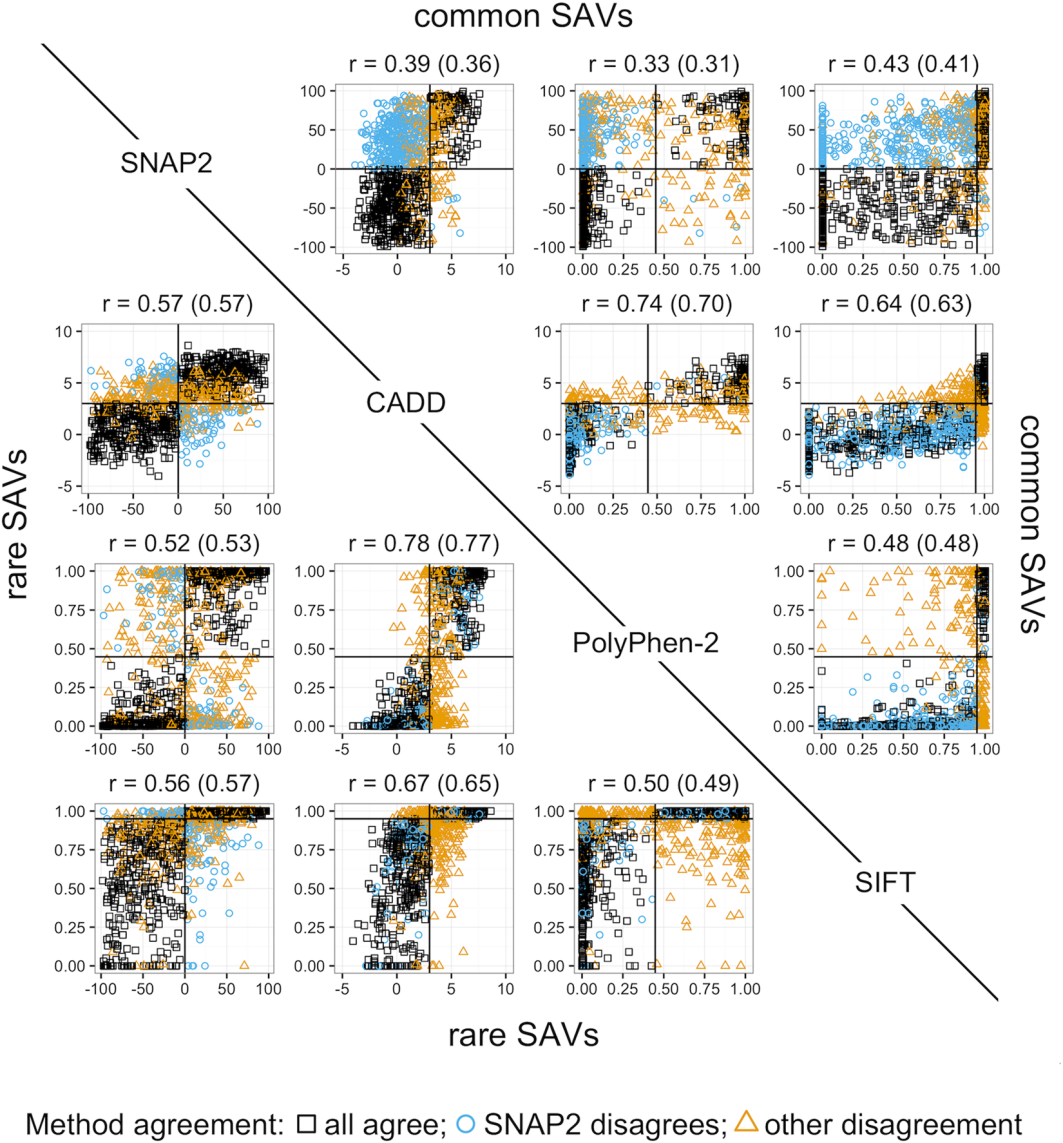
Methods correlated more for rare than for common 1KG SAVs. Each plot shows the correlation of functional effect scores between one pair of prediction methods for two samples of 1,000 rare and 1,000 common SAVs from 1KG. Results for common SAVs are shown above diagonal, those for rare SAVs are given below the diagonal. With the order of the plots being 1 = SNAP2, 2 = CADD, 3 = PolyPhen-2, and 4 = SIFT, this implied that the plot corresponding to matrix element Pmn compared common SAVs between methods m and n (above diagonal), and the element Pnm rare SAVs between those two (below diagonal). For instance, row = 1/column = 2 gave the correlation between SNAP2 and CADD for common SAVs, while the transposed element row = 2/column = 1 correlated rare SAVs for SNAP2 and CADD. Each point represents a pair of scores for a single SAV, e.g. from SNAP2 and CADD. The predicted score for SIFT has been inverted (1-SIFT) to ease the comparisons. The shape and color reflect the overall method agreement. We use the following code: black squares mark SAVs for which all four methods agree on the binary classification. Blue circles mark SAVs for which all methods but SNAP2 agree; orange triangles mark all other points. The Pearson Correlation Coefficient for all 1,000 SAVs was added above each plot, along with the corresponding value for the full set of all SAVs (in brackets, as in Table 2).

SNAP2 predicted a similar fraction of rare SAVs (LDAF < 1%: 209,928 variants, *i.e*. 81% of all *1KG*) to have an effect as did PolyPhen-2^16^, CADD^17^, and SIFT^18^ (Table 1; range from 40–50%). This statement comes with the caveat that the binary classification requires the introduction of a threshold (effect/neutral) that may not be appropriate for a particular tool. For instance, for CADD, and to a large extent for SNAP2, the particular threshold used depends on the data set and the question being asked. Overall, the predictions for rare SAVs also correlated pairwise between SNAP2, PolyPhen-2, CADD, and SIFT (Table 2, Fig. 5: below diagonal: correlations from 0.49–0.77). However, the set of rare SAVs for which all four methods predicted an effect was around 28% (data not shown), *i.e*. the methods correlated on average, but differed in detail in the trends captured.

**Table 1:** Similar ratios of neutral/effect predicted by four methods for 1KG SAVs*.

SAV set		SNAP2*	CADD	PolyPhen-2	SIFT
effect/all	common	61%	19%	18%	19%
	rare	50%	50%	42%	40%
	all	51%	46%	39%	47%
neutral/all	common	39%	81%	82%	81%
	rare	50%	50%	58%	60%
	all	49%	54%	61%	63%

*Data sets: Rare SAVs (LDAF < 0.01): all methods agreed within ten percentage points on the ratio; common SAVs (LDAF ≥ 0.05); the values for “all” also included uncommon SAVs (0.01 ≤ LDAF < 0.05). Methods: SNAP2* implies error-corrected estimates for SNAP2 (below). The four methods compared here have different aims and use different thresholds. Here, we applied defaults to simplify the comparison and interpreted predictions as binary (effect/neutral) according to those thresholds. In particular, we chose the following thresholds. Effect: SNAP2-score > 0, CADDv1.3 raw score > 3, PolyPhen-2 = *probably or possibly damaging*, SIFT = *deleterious*; neutral: SNAP2-score ≤ 0, CADDv1.3 raw score ≤ 3, PolyPhen-2 = *benign*, SIFT = *tolerated*. SNAP2* error correction: Values for SNAP2 were corrected for false positives and false negatives (e.g. Neff* = Neff(raw) − FPR(Neff(raw)) + FNR(Nneu(raw)). Error correction lowered the estimates for common effect and increased that for rare effect.

**Table 2:** Predictions more correlated for rare than for common 1KG SAVs*.

	SNAP2	CADD	PolyPhen-2	SIFT
SNAP2		0.36 ± 6.8*10^−3^	0.31 ± 6.9*10^−3^	0.41 ± 6.6*10^−3^
CADD	0.57 ± 1.8*10^−3^		0.70 ± 5.2*10^−3^	0.63 ± 5.7*10^−3^
PolyPhen-2	0.53 ± 1.9*10^−3^	0.77 ± 1.6*10^−3^		0.47 ± 6.4*10^−3^
SIFT	0.57 ± 1.8*10^−3^	0.65 ± 1.7*10^−3^	0.49 ± 1.9*10^−3^	

*Pearson correlation coefficients above the diagonal show the agreement for common 1KG SAVs, those below the diagonal for rare 1KG SAVs. Correlation values were calculated using the predicted raw scores of SAVs for which predictions were available for each method. The correlation in predictions between the four methods was higher for all pairs below the diagonal, *i.e*. for rare SAVs. Standard error of r: SE_r_ = sqrt((1 − r^2^)/(n − 2)); n_common_ = 18,876; n_rare_ = 209,928.

PolyPhen-2, CADD and SIFT also largely agreed in their classifications for the overall amount of effect/neutral for common SAVs (LDAF ≥ 5%: 18,876 variants, *i.e*. 7% of all *1KG*, Fig. 5: above diagonal, agreeing predictions in lower left and upper right quadrants). In contrast SNAP2 predicted larger fractions of the common than of the rare SAVs to have an effect*, i.e*. SNAP2, on average, predicted stronger effects for common than for rare SAVs (Fig. 4: *common* moved toward upper right, *i.e*. more effect). The shift could also be expressed by calculating the “effect AUC” of the density distribution for SNAP2 scores, *i.e*. the Area-Under-the-Curve (AUC) from 0 ≤ SNAP2-score ≤ 100: AUC = 0.61 for common and AUC = 0.48 for rare SAVs (Supplementary Fig. S4). Another example highlighting the difference between common and rare SAVs: over 12% of the common, as opposed to fewer than 6% of the rare, SAVs were predicted to affect function as strongly (same SNAP2-score) as 50% of the strongest OMIM SAVs (Fig. 1 *OMIM*).

### SAVs with effects not distributed randomly

We investigated whether or not SAVs predicted with strong effect (SNAP2-score >50) were randomly distributed through two analyses: simple statistical enrichment and the enrichment of sub-cellular localization.

Firstly, we investigated the distribution of common SAVs per protein. For about 18k proteins the 60KE data set annotated rare and common SAVs in the same protein. For 1,254 of those there was more than one common SAV and SNAP2 strongly predicted over 50% of the common SAVs to have effect (SNAP2-score >50). An analogous filter on the rare SAVs (≥2 rare SAVs in same protein + ≥50% of rare SAVs at SNAP2-score >50) led to only 304 out of 18k proteins. Furthermore, nine proteins (ENSP00000363436, ENSP00000369568, ENSP00000408146, ENSP00000337397, ENSP00000363433, ENSP00000353078, ENSP00000415517, ENSP00000310338, ENSP00000413079) display ten or more common variants, and more than 50% have a SNAP2-score >50. All those nine proteins contain more rare variants with SNAP2-score ≤50 than with SNAP2-score >50.

Secondly, we predicted the sub-cellular localization for all proteins with LocTree3^19^ (given the many annotations available for human proteins, most predictions were based on homolog-inference). Of all the proteins with SAVs, ~28% were predicted to be nuclear and about ~23% as secreted or cell membrane. We compared these numbers to different ways of scanning for proteins enriched in effect predictions. For the subset of all proteins with at least 50% of their residue positions observed as a SAV, and 50% of those predicted with SNAP2-score >50, 17% were nuclear (reduction to 60% of expected) and 40% secreted or cell membrane (1.7 fold over-representation over expected. We found a similar over-representation of “secreted + cell membrane” in proteins with many effect SAVs when looking at the subset of all proteins with at least 4 common SAVs for which at least 30% were predicted at SNAP2-score >50 (value of 30% guided by the average expected at that SNAP2-score, cf. Fig. 4-common). About 44% of those proteins were predicted as secreted or cell membrane, i.e. 1.9 times more than expected. Finally, only 29 proteins were so enriched in common SAVs that 10% of all residues had common SAV. LocTree3 predicted 21 to be secreted.

## Discussion

### Prediction distributions are different for sets of SAVs

We analyzed sets of SAVs that were predicted to have effect or not. To estimate the number of expected mistakes, we have to provide extensive performance evaluations of our method. Instead, of these approximations we compared distributions of different sets of SAVs and their predicted effects. Even with relatively high rates of errors, methods can set such distributions reliably apart as long as their mistakes are not systematic with respect to the results. Below we argue that such systematic bias explains the differential results for prediction methods with respect to human SAVs that are common and rare. For instance, if we measured the height of women in Greece and Germany, we might find out that they differ by barely 2 cm, while the standard deviation for the measures are 5-times this difference. Despite the considerable standard deviation, we can easily distinguish between two countries of millions of individuals. The same is true for the differences between the distributions for the prediction of SAV effects.

### Cross-species vs. 1KG and 60KE: changes from a point frozen in time

The cross-species “SAVs” appear to be describing something very different from the SAVs in the contemporary human population (1KG/60KE sets). Our analysis, nevertheless, assessed the effects of sequence variation in the same way. Our first results (Fig. 1A) compared orthologous proteins between human and other species; i.e. each curve was based upon a different set of proteins–the subset of proteins orthologous between human and the other species. We chose these comparisons because using the subset of proteins common to all species is so small that is clearly not representative of all proteins, a set that we might refer to as the “house keeping” proteins. Any results obtained exclusively for these specially selected proteins might be biased. Surprisingly, the conclusions did not change between taking “all orthologs” and restricting the analysis to “house keeping” genes (Fig. 3 dashed vs. solid lines). In fact, by filtering the data differently, we could even confirm the major trends to a level as far diverged as human-fly (Supplementary Fig. S3).

How can SAVs between people affect function more than those between species? We compared SAVs within a dynamic, evolving population (human) with SAVs that describe a speciation event, *i.e*. were frozen in time. At speciation, SAV effects between descendants likely randomly sampled the 1KG data. Thereafter, each protein carrying a “speciation SAV” had two possible fates. Either, the protein has drifted away in sequence and function to an extent that it has not been considered in our comparison of evolutionarily related proteins (orthologs). Or, it has maintained function by constraining variation to neutral SAVs. Thus, inter-species SAVs might appear to be more neutral with increasing evolutionary distance due to the process of removing proteins with too many effect SAVs from the comparison. Indeed, the subset of “house-keeping” proteins differed substantially in their effect from all proteins (difference between dashed and solid lines in Fig. 3 and Supplementary Fig. S3). However, the reported effect that 1KG SAVs were predicted, on average, with more effect than cross-species SAVs was valid both for only “house keeping” and for “all proteins”. This signal could therefore neither be explained by data bias nor by data inconsistencies.

### Will all possible SAVs be observed?

Not all possible SAVs have been observed in healthy people^3^: some may be lethal and prevent the carrier from being sequenced; others might prevent development in much earlier stages, even long before birth. Will we observe all remaining ones? Our *in silico* mutagenesis experiment randomly sampled impact predictions for every possible SAV (19-non-native Supplementary Fig. S2) and every SNV-possible SAV (SNV-possible in Supplementary Fig. S2). This enabled us to gauge the expected effect of random variation in comparison to those observed in 1KG. We noted small but significant differences between what has been observed between healthy people and a random subset (Supplementary Fig. S2). This implied that the SAVs observed in 1KG were NOT random subsets of all possible SAVs. This in turn enabled the estimation of what to expect from observing SAVs for a larger population.

When we analyzed the data for the 60KE set, we confirmed the above observation; namely that the score distributions for rare SAVs approached random (SNV-possible, Fig. 4). Although the difference between random and rare became smaller, it remained significant (p-value < 2.2e-16). There will always be a difference between what can be observed and what can be simulated: some SAVs will simply be “too fatal” to observe^20^. With the 60KE curve for rare SAVs so close to random, did this imply that such an effect was relatively minor, *i.e*. that very few SAVs are that deadly? Answers remain speculative. One problem lies in the scale of the numbers: even if thousands of SAVs were deadly, their effect could easily be overshadowed by >7 million SAV set size.

### Common SAVs affect function more than rare SAVs?

Many colleagues who we have confronted with our data had expected the predictions for rare SAVs (observed in <1% of population) to be moved toward “more effect” (moved toward right in Fig. 4) than those for common SAVs (observed in >5% of the population). Predictions from CADD, PolyPhen-2, and SIFT confirmed this expectation (Supplementary Fig. S5), while SNAP2 predicted the opposite, *i.e*. predictions for common SAVs were shifted toward “more effect” than for rare SAVs (Fig. 5). We also established that SNAP2 on the one hand and CADD, PolyPhen-2, and SIFT on the other hand largely agreed on the fraction of effect/neutral predictions for rare SAVs (Tables 1 and 2). Although all four methods agreed for only about half of effect predictions for the rare SAVs, two-method correlations were fairly high for rare SAVs (Table 2, lower diagonal).

Could SNAP2 predictions be wrong more often when they disagree with three other methods that agree with each other? First off: CADD, Polyphen-2 and SIFT agreed with each other more for rare than for common SAVs (Table 2, values higher on lower than on upper diagonal). Thus, the view of “three over one” is not fully supported by the details.

The extreme hypothesis is that for the majority of common SAVs for which SNAP2 predicts more effect than the other methods, SNAP2 is wrong. We have published two findings that clearly refuted this hypothesis for two of the methods (PolyPhen-2 and SIFT). Firstly, all three methods performed significantly worse for difficult-to-predict SAVs. Secondly, for a subset of human SAVs that had been used to train PolyPhen-2 and was biased by a predominance of effect, SNAP2 still predicted much better than SIFT (Q2-SNAP2 = 58% vs. Q2-SIFT = 44%) and *on par* with PolyPhen-2 although the latter had the advantage over SNAP2 of having been optimized on these data^21^. Although these results suggest that SNAP2 was right for most of the SAVs for which it’s prediction differed from the others, SNAP2 might still systematically mis-predict common SAVs.

Could details in methods development result in systematic mistakes for common SAVs? SNAP2 was developed predominantly on rare SAVs. More explicitly, of all the SAVs trained to have effect, about one quarter were *OMIM-like*, *i.e*. resembled rare SAVs. Thus, SNAP2 is likely biased towards predicting rare SAVs as having more effect than common. Correcting for this bias, we expect the “true” common curve to be moved even more to the right (towards more effect). In contrast, PolyPhen-2, CADD, and SIFT have been built using principles that might explain the bias toward “SAVs common to a population neutral”. PolyPhen-2 was optimized to differentiate between monogenic disease-causing SAVs (rare by definition) and variation between orthologs. This implies that the machine learning may have enforced a much more substantial and explicit bias toward “rare have effect” by teaching “100% of the effect is rare”, as opposed to “25%” for SNAP2. CADD implicitly shares some of this bias as it optimizes the separation between simulated variants and variants that differentiate orthologs. The simulated variants, by definition, are not observed, most likely because they cause disease or are even lethal, *i.e*. impact the whole organism. SIFT is built upon a similar idea, namely that SAVs conserved throughout evolution are likely to be neutral, suggesting that common effect are systematically unlikely to be captured by this method. While SNAP2 also uses conservation, many of the SAVs used for training SNAP2 *effect* were not conserved; conversely, many SAVs used to train *neutral* were conserved. Thus, the neural networks underlying SNAP2 might have put SNAP2 into the unique position of correctly spotting SAVs that are conserved, yet might affect function^21^.

The differences in bias in the training data between SNAP2/SNAP1 on the one side and between PolyPhen-2, CADD, and SIFT on the other side might explain why common SAVs were predicted so differently. A related explanation pertained to different objectives. SNAP aims at predicting the effect of SAVs upon protein function; most training data measures molecular function rather than biological process. In contrast, PolyPhen-2, CADD, and SIFT, have been optimized with a more focused view upon pathogenicity for an organism. SNAP2 might correctly identify trends in the changes of molecular function because it avoids labeling variants in the context of the pressure exerted upon the organism.

SNAP2 cannot distinguish between SAVs that help and those that hurt the organism^15^. Although common variants might affect molecular function significantly, as suggested by SNAP2, they are unlikely to cause severe diseases, as illustrated by the predictions of PolyPhen-2 and CADD, since such extreme functional disruptions are unlikely to spread in a population. Nevertheless, SNAP2, CADD, PolyPhen-2, and SIFT predicted similar ratios for neutral/effect for rare SAVs and are fairly correlated for those (Tables 1 and 2). The big disparity that flips the prediction from “common less effect” to “rare less effect” between SNAP2 and the others is based only on the common SAVs.

An alternative way to explain the difference between SNAP and the other three (CADD, PolyPhen-2 and SIFT) is the following hypothesis. (1) All methods might capture the effects of monogenic-disease causing SAVs of the OMIM-type equally well, and therefore agree in their predictions for rare SAVs. (2) Effects for common SAVs are less likely to impact the organism although they might strongly affect molecular function, and SNAP2 might be the best of the four methods in capturing such effects upon molecular function.

To prove this hypothesis, we have to show that many common SAVs have effects and have never been used by any of the methods for training. Unfortunately, there is no experimental data available to support or refute this point. However, recent deep-scanning experiments might at least show trends as to which method is best at capturing effects upon molecular function. In one case study, we compared the correlation between CADD and SNAP for one particular protein^22^ (BRCA1 in Supplementary Fig. S6). Hopf *et al*. have recently analyzed the performance of prediction methods for several deep-scanning experimental data sets^23, 24^. This analysis confirmed the trends that we observed: SNAP2 captured effects upon molecular function better than the other three methods. Incidentally, those analyses also suggested that the low-throughput experiments previously used to develop and assess prediction methods might over-estimate performance, at least if taking high-throughput deep-scanning experiments as a better proxy for reality. Thus, while many SNAP2 predictions might be wrong, all analyses converge upon the explanation that SNAP2 captures the effect of common SAVs upon molecular function better than CADD, PolyPhen-2, or SIFT. If so, the SNAP2 predictions discovered an unexpected reality, namely that common SAVs have, on average, more effect upon molecular function than rare SAVs. Common SAVs, in fact, might be relatively enriched through evolutionary selection as those that affect molecular function in a way that might help to drive the evolution of the species.

## Conclusion

Our results are compatible with the distinction of two types of Single Amino acid Variants (SAVs). (1) *Point punches*, *i.e*. genetic alterations leading to large molecular changes that significantly diversify proteins and molecular pathways of individuals. (2) *Additive small effects*, *i.e*. near-neutral variants in many genes whose cumulative interplay is responsible for the impact upon pathway-wide molecular functionality. In this view, isolated strong-effect SAVs usually do not drive speciation. Within the individuals of one species, however, many of the common variants strongly impact protein function for better or worse (gain- vs. loss-of-function). Together, these findings might imply that common SAVs are unlikely to drive speciation. How much variation is beneficial to the individual and how much is necessary for the survival of the species? To answer this conundrum, we need better experimental and computational tools that distinguish the directionality of change and bridge from the micro-molecular view of single sequence variants to the macro-systems view of phenotypic impact for the organism.

We also observed that although *in silico* methods often agree in the effects they predict for SAVs, their differences are substantial enough to completely invert predicted trends as extreme as from “common SAVs have more effect” to “rare SAVs have more effect”. We argued that such crucial differences originated from the way the methods were trained, and that SNAP2 picked up a crucial aspect of molecular function that were missed by others. While it remains unclear how the impact of SAVs upon molecular function translates to the impact upon the organism, the inference from the micro- to the macro-level will remain obfuscated. Specialized *in silico* methods combined with experimental deep scanning might bring about more clarity in the future. Until then, we remain with very surprising findings for the impact of sequence variation upon molecular function.

## Methods

### Data variants (SAVs)

Our work focused entirely on sequence variants that alter a single amino acid in the protein. We referred to those as **SAV** (single amino acid variant; abbreviations found in the literature for the same include: nsSNV, nsSNP, and SAAV). We analyzed the following subsets separately.

#### OMIM

Set of disease-causing variants reported in OMIM^4^ as extracted from SNPdbe^25^. SNAP2 scores were calculated for 5,661 SAVs in 1,547 unique protein sequences. The scores reported for OMIM in all figures were calculated with a special version of SNAP2 trained without using OMIM and HumVar^16^ SAVs. Additionally, we generated a second set of SNAP2-scores for the above mentioned 5,611 OMIM SAVs through cross-validation. For this purpose, we retrained SNAP2 on the full training set (including OMIM and HumVar SVAs) holding out a small subset of OMIM SAVs as test-set in each iteration of the cross-validation (Supplementary Fig. S1).

#### 60KE

SAVs reported by the Exome Aggregation Consortium (ExAC) at the Broad Institute reporting SAVs for 60,706 exomes^3^. We extracted all SAVs from ExAC release 0.3.1 labeled as ‘missense_variant’ and ‘SNV’ in the ‘CSQ’ information field. The resulting total was 10,474,468 SAVs; for 7,599,572 of these SNAP2 could predict the impact on molecular function. 40,446 were classified as common (LDAF ≥ 0.05), 28,789 as uncommon (0.01 ≤ LDAF < 0.05), and 7,530,337 as rare (LDAF < 0.01).

#### 1KG

SAVs in human reported by the 1000 Genomes Project^12, 13^. In particular, we included SAVs labeled as NON_SYNONYMOUS retrieved from the CADDv1.3 dataset^17^. 1KG variation in CADDv1.3 is based on 1000 Genomes Project Phase1 release v3.20101123. This set contained 292,848 variants, of which SNAP2 scores could be obtained for 268,115 SAVs (91.6%) in 19,696 sequence-unique proteins. Almost all missing SNAP2 predictions originated from problems with the underlying SIFT runs. Less than 1% of the SAVs were not predicted due to the SNAP2 limitation to exclude proteins with over 6,000 residues. Out of the 268,115 variants 20,352 were classified as common (LDAF ≥ 0.05), 30,543 SAVs as uncommon (0.01 ≤ LDAF < 0.05), and 217,220 variants as rare (LDAF < 0.01).

#### Inter-species orthologs

Orthologs were extracted from ENSEMBL Genes 73 entries (release Sep. 2013) using the Biomart^26^ interface. To determine homology, ENSEMBL uses EnsemblCompara GeneTrees^27^. Pairs of human proteins (hg19) and other species’ orthologous proteins were aligned using the Needleman-Wunsch EMBOSS implementation^28^ with default parameters (BLOSUM 62, gap open = 10, gap extend = 0.5). All alignments with PIDE < 70% (excluding gaps) were discarded. For all remaining pairs of aligned proteins, every amino acid variant was considered a SAV. Each SAV was evaluated in the context of the non-human sequence such that the residue position with a difference was mutated to the human amino acid. For multiple orthologs of the same protein (*e.g*. FoxP in Fly to FoxP1/2/3 and 4 in human) the highest ungapped sequence identity alignment was chosen to extract the SAVs. We avoided bias by excluding all variants that were used for SNAP2 training. The numbers of SAVs in resulting sets were as follows: chimp – 95,624 SAVs in 14,361 proteins; mouse – 379,795 SAVs in 12,616 proteins; fly – 16,403 SAVs in 364 proteins.

#### “House-keeping”

This was a subset of all proteins that were considered as orthologs in cross-species comparisons (human-X). For the main figure (Fig. 3), we compared predictions for human-chimp variants, human-mouse variants and 1KG SAVs to the subset of variants from orthologs common to all species. Additionally, we further restricted the comparison to proteins for which SAVs were available in all three orthologs of the protein in three species (e.g. excluding cases for which human-chimp had no SAVs between protein X_human_ and X_chimp_, while human-mouse had SAVs between proteins X_human_ and X_mouse_). Number of SAVs in resulting sets: human–133,500 SAVs in 8,535 proteins; chimp 46,288 in 8,147 proteins; mouse 309,516 SAVs in 8,235 proteins.

#### Denisovan

Amino acid differences between *Homo sapiens* and *Denisova hominin* were extracted from the data published by the groups of Svante Pääbo and Janet Kelso^29^. In our implementation, the *denisovan* amino acid (ancestor) was introduced into the corresponding position in the human protein and then mutated back to the human reference. For example, if the human sequence Xp contained amino acid L at position 42 and the corresponding *denisovan* residue was V, we created a protein sequence Xp’, equivalent to Xp except for V at position 42. For simplicity, we referred to these variants as SAVs because technically they originated from the same “edit procedure”, i.e. the change of a single amino acid. We then predicted the effect of the Xp’ SAV V42L. This set contained 236 SAVs in 292 proteins.

#### Random

We created two ‘random’ sets of human SAVs. Both sets are a random sample (n = 268,511) of two ‘supersets’: (1) all 19-non-native SAVs; (2) all SNV-possible SAVs, i.e. SAVs that can be reached by mutating one single nucleotide, which is in turn a subset of (1). The size of the random sample was chosen to be 268,511 to be in line with the size of the 1KG SAV set.

## Methods

Effect scores for SAVs in all sets were computed using SNAP2^21^, an improved version of SNAP1^15^. SNAP2 uses a protein sequence and a list of SAVs as input to predict the effect of each substitution on the protein molecular function. The prediction scores range from −100 for fully neutral to +100 for strong effect. In its original form, SNAP scores served only as reliability index, where the confidence that the assigned class (neutral/effect) for a specific mutation is higher for SNAP scores closer to the −100 and +100 maxima. However, the score also correlates clearly with the severity of the effect^15^, i.e. scores slightly above 0 are not as severe as those closer to +100. Interestingly, for mis-predicted neutral SAVs (i.e. SAVs with known effect, incorrectly predicted as neutral), the scores closer to 0 indicate higher than for those scoring closer to −100. For all SAVs in all sets, SNAP2 scores were computed.

For a binary projection (effect/neutral), SNAP learned to optimize the experimental annotations such that SNAP2-score ≤0 implied neutral and SNAP2-score >0 implied effect. The experimental evidence for effect is much more reliable than evidence for neutral. Therefore, the point that optimally fits the known data might not describe reality best^21^. This discrepancy calls for introducing additional thresholds for the binary distinction effect/neutral. All of those are arbitrary, i.e. are meaningful only to highlight trends. The raw data is the full spectrum of the prediction (−100 to +100). Therefore, we showed this full spectrum in all figures.

To simplify the communication of trends, we defined three example thresholds for SNAP2 in addition to the default of 0: (1) SNAP2-score ≤−42 (below this point we expect 85% of all predictions to be correctly predicted as neutral, Fig. 1– lower panel), (2) SNAP2-score ≥+50 (above this point we expect 85% of all predictions to be correctly predicted as effect, Fig. 1– lower panel), (3) SNAP2-score ≥+75 (chosen because above this point 50% of all OMIM SAVs are correctly predicted; effect prediction accuracy of 88%). Accuracy values are based on the number of variants that are reported above (for effect) and below (for neutral) the respective thresholds, i.e. for the effect prediction accuracy at the threshold ≥+50 SAVs with predicted SNAP2-scores between +50 to +100 were considered and are predicted correctly with an accuracy of 85%.

### Other methods

PolyPhen-2 ^16^ measures the likelihood whether a SAV is pathogenic or benign based on family conservation and structural information. The method has been trained on disease-related SAVs from HumDiv and HumVar, and on SAVs between orthologs in human and mammal considered as neutral. SIFT ^18^ uses family conservation to measure the probability for a SAV to be deleterious or tolerated (as expected from alignment). CADD^17^ aims at distinguishing between “variants that survived natural selection” and simulated mutations. CADD has been trained on mutations between an inferred human-chimp common ancestor and the human reference genome (excluding common SAVs in 1KG; however, including those where the reference allele carries the ancestral variant and the derived 1KG allele occurs in more than 95% of the population). Simulated variants are created through a process of mutating nucleotides based on parameters extracted from the inferred human-chimp ancestor and sequence alignments between multiple primate species. Scores and classification for PolyPhen-2, CADD and SIFT were extracted from the CADDv1.3 data set. PolyPhen-2 effect scores were available for 260,039 SAVs (common: 19,272 - uncommon: 29,281 - rare: 211,486). CADD scores were available for all SAVs. SIFT scores were available for 264,565 SAVs (common: 19,704 - uncommon: 29,898 - rare: 214,963).

### Estimated method performance

SNAP2^15, 21^ has been estimated to perform at a sustained positive accuracy (TP/(TP + FP)) 78% and a negative accuracy (TN/(TN + FN)) of 77% (at the default SNAP-score of 0, Fig. 1 lower panel). Prediction accuracy rises with increasing thresholds (effect: toward SNAP-score +100 on the right in Fig. 1; neutral: toward SNAP-score −100 on the left). For the example thresholds SNAP2-score >+50 and SNAP2-score >+75 the accuracy of predicted functional effect increased to 85% and 88% respectively, whereas at SNAP2-score <−42 the expected accuracy for predicting neutral increased to 85%.

#### From molecular function to disease

SNAP1 correctly predicted over 80% of the SAVs for which a monogenic disease (OMIM^4^) was known four years ago (1,105) at its default threshold^25^. For this work, we repeated this analysis with a fivefold larger set of monogenic disease-causing variants in OMIM (5,611) using a version of SNAP2 that had not used OMIM-type of variants for training at all (Fig. 1 *disease*). In fact, for all versions of SNAP the OMIM SAVs that had not been used for method development were predicted at much higher average scores than variants in our training set^21^. The SNAP2-score primarily serves as a reliability index. However, we previously demonstrated^6, 15^ that effect strength also correlates well with the SNAP-score, e.g. more reliably predicted effect SAVs tend to have stronger effect.

### Standard error estimates

The standard errors of the mean were estimated by bootstrapping the SNAP2-scores of the respective datasets. The datasets were resampled with replacement a hundred times, calculating 100 means of the SNAP2-score distribution. Standard error was estimated as the standard deviation of the means.

## Electronic supplementary material

Supporting Online Material
