# Supplementary material for: Common sequence variants affect molecular function more than rare variants?
Source: Sci Rep. 2017 May 9;7:1608. doi: 10.1038/s41598-017-01054-2 (PMC5431670; doi:10.1038/s41598-017-01054-2)
Supplement: Supplementary file 1 — Supporting Online Material [file 41598_2017_1054_MOESM1_ESM.pdf]

Supporting online material (SOM) for:
Common sequence variants affect molecular function more than rare variants?

Yannick Mahlich [1, 2, 3*], Jonas Reeb [1], Maximilian Hecht [1], Tjaart Andries Petrus De Beer [4], Yana Bromberg [2, 3] & Burkhard Rost [1, 3, 5]

1 Computational Biology & Bioinformatics - i12, Informatics, Technical University of Munich (TUM), Boltzmannstrasse 3, 85748 Garching/Munich, Germany
2 Department of Biochemistry and Microbiology, Rutgers University, New Brunswick, NJ 08901, USA
3 Institute of Advanced Study (TUM-IAS), Lichtenbergstr. 2a, 85748 Garching/Munich
4 European Molecular Biology Laboratories, European Bioinformatics Institute (EMBL-EBI), Welcome Trust Genomes Campus, Cambridge, Cambridgeshire, United Kingdom
5 Institute for Food and Plant Sciences WZW – Weihenstephan, Alte Akademie 8, Freising, Germany
* Corresponding author: Yannick Mahlich (ymahlich@bromberglab.org)

Table of Contents for SOM

Fig. S1 – Training on disease-causing SAVs improves prediction for those ...… p. 2

Fig. S2 – 1KG SAVs differ from random SAVs ……………………………………. p. 3

Fig. S3 – Orthologs across four species same trend as entire proteomes ...…... p. 4

Fig. S4 – SNAP2 predicts more common than rare SAVs to be effective ……… p. 5

Fig. S5 – CADD, PolyPhen-2 and SIFT predict a higher fraction of rare variants to be functionally effective than common variants …………………………………… p. 6

Fig. S6 – SNAP2 captured molecular function better than CADD for deep scanning BRCA1 dataset …………………………………………………………………...... p. 7

SOM Note – SNAP2 training data ……………………………………………….. p. 9

References for SOM ………………………………………………………….... p. 10

Material

Fig. S1:

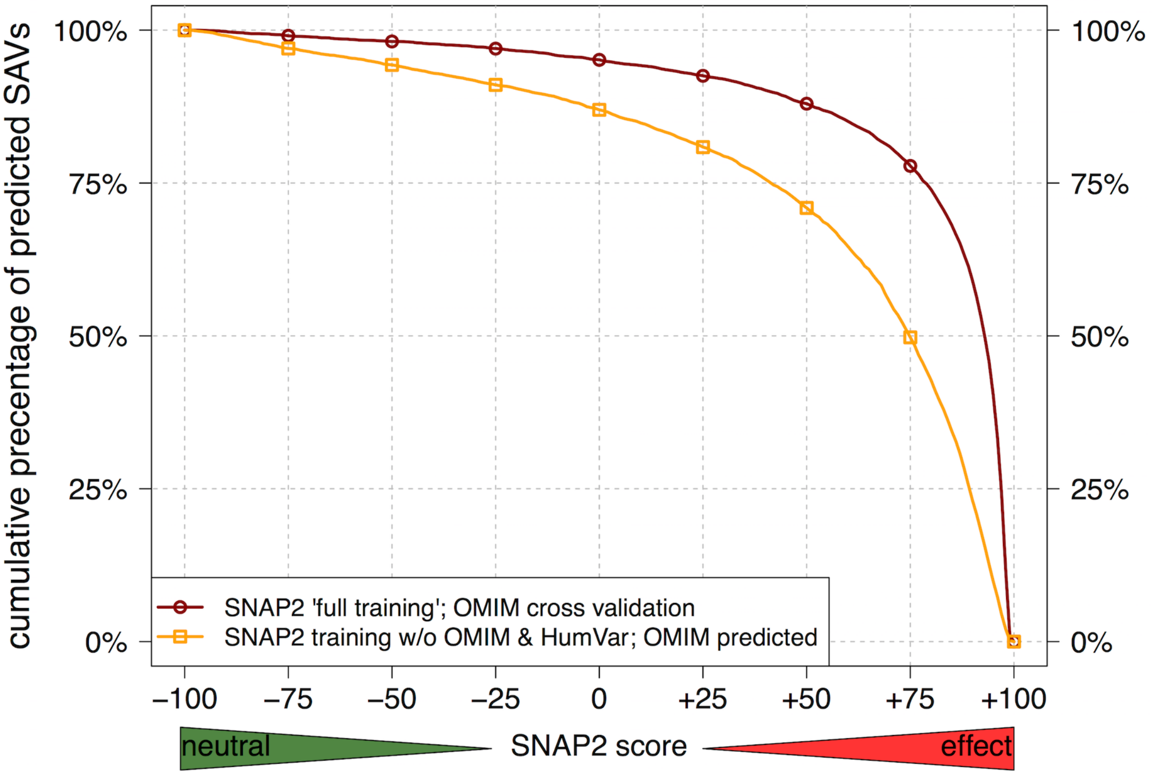

Fig. S1: Training on disease-causing SAVs improves prediction for those. The curves refer to monogenic SAVs from OMIM directly implied in disease (c.f. Method section in main manuscript). Here, we compared two different SNAP2 versions: the first (dark red, circles) was trained using SAVs from OMIM and HumVar. Results were obtained through cross-validation, i.e. the SAVs shown here were not in proteins sequence-similar (HVAL>0 AND PSI-BLAST EVAL<10-3) to proteins used for training. In contrast, the SNAP2 version labeled “SNAP2 training w/o OMIM & HumVar” (orange, squares) never used any disease-impact SAV for training. The difference between the two versions of SNAP2 demonstrated how much training on disease-causing SAVs helps to predict (dark red much higher than orange). In turn this suggested that methods trained on features relevant to disease-causing SAVs capture different aspects than methods not using such SAVs.

Fig. S2:

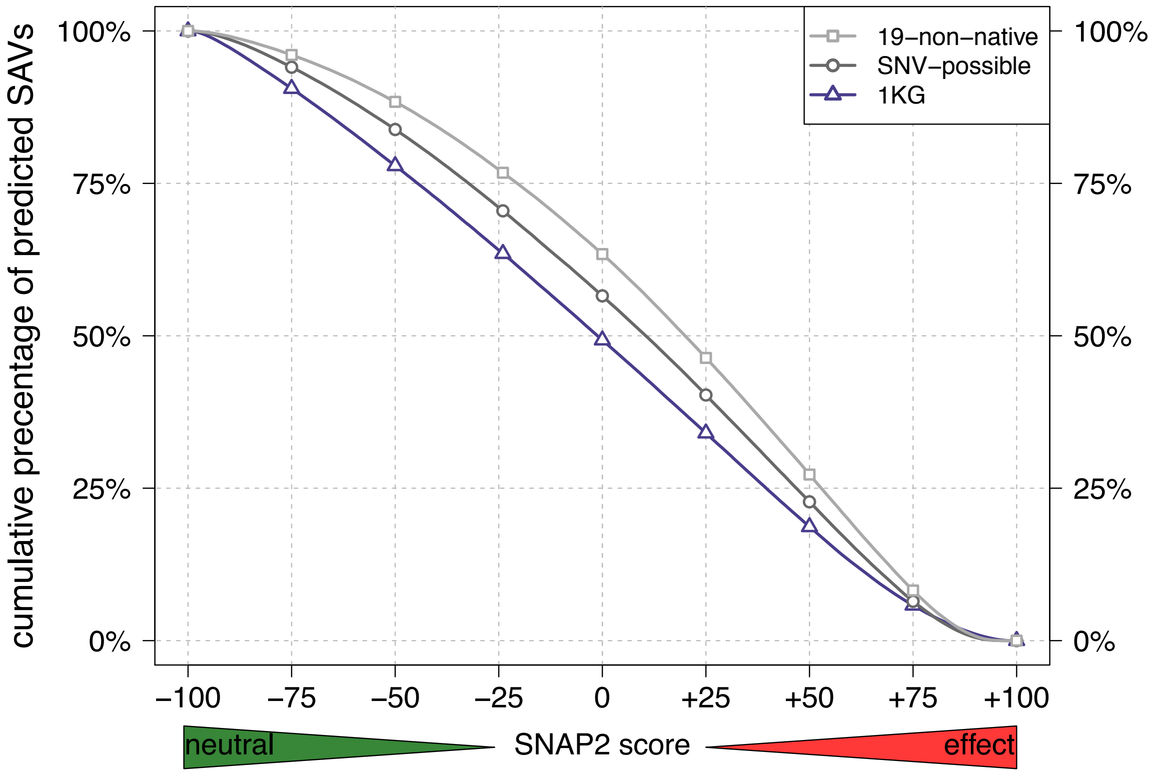

Fig. S2: 1KG SAVs differ from random SAVs. All curves show predictions from the standard SNAP2 version. We compared the predictions for all SAVs in the 1KG set of healthy people (blue curve, triangles) to random subsets of all possible SAVs that we could generate *in silico*. We have two options to realize "all possible": replace all native amino acids at all residue positions by (1) all 19-non-native amino acids ("19-non-native"), and (2) all amino acid substitutions that can be reached by a single nucleotide variant ("SNV-possible"). We then selected a subset of these large data sets with the same number of SAVs as in the 1KG set. This gave the sets random-19-non-native (light gray, squares) and random-SNV-possible (dark gray, circles). Although the difference between all curves appeared to be minor, they were statistically significant. We tested the significance by the two-sample Kolmogorov-Smirnov (KS) test (1KG vs. SNV-possible, D = 0.072; 1kg vs. 19-non-native, D = 0.141; 19-non-native vs. SNV-possible, D = 0.069; n,n' = 268115, estimated p-value < 2.2e-16 for all three KS-tests). Since the standard error of mean (SEM) for all three SNAP2 score distributions was < 0.5, error bars and confidence intervals were omitted.

Fig. S3:

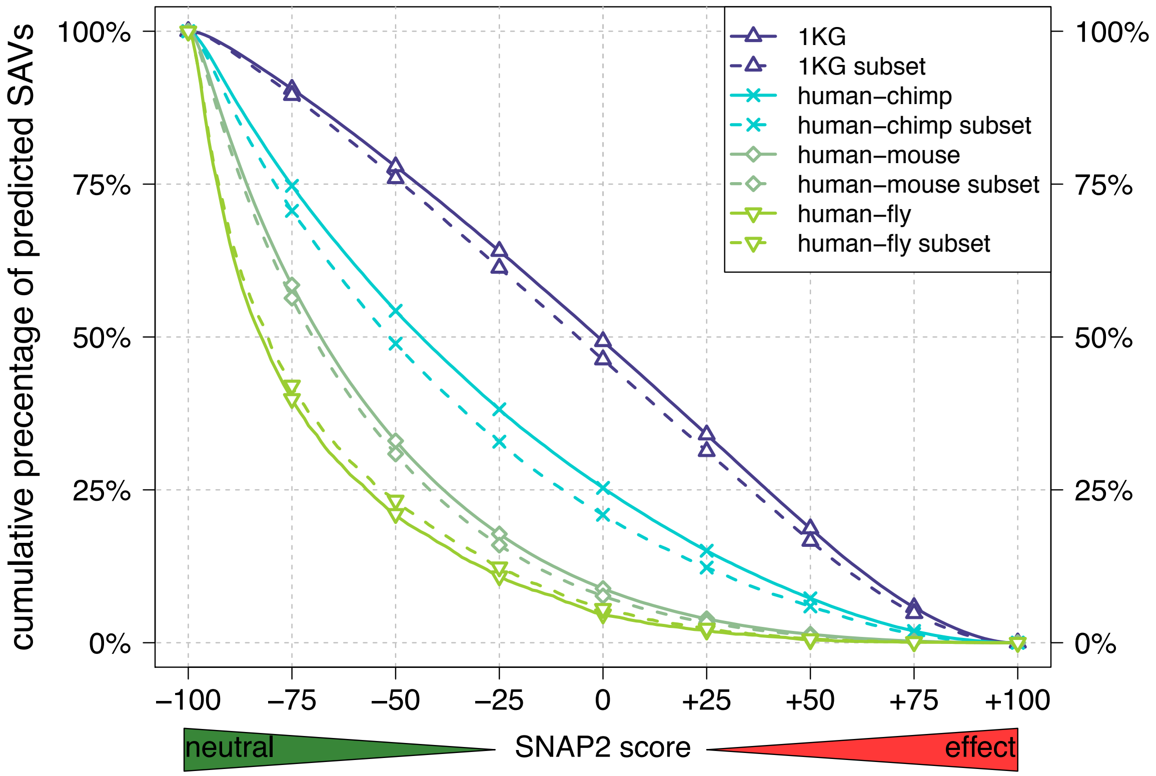

Fig. S3: Orthologs across four species same trend as entire proteomes. While Fig. 1 in the main manuscript compares cross-species and 1KG SAVs for all proteins (and thereby compares different sets of proteins), and Fig. 2 compares a much smaller data set of orthologs shared between three organisms and 1KG, here we compiled results for SAVs that is less restrictive. The main restriction, that a protein used to extract SAVs has to have an ortholog in the other species still applies, however, we do not exclude proteins for which in one or more of the human-X inter-species comparisons do not yield SAVs. To give an example: The orthologous proteins X_{human}, X_{chimp}, X_{mouse} (and X_{fly}) are only considered for SAV extraction in Fig. 3 if all proteins contain SAVs. Here we include SAVs from those proteins into the analysis even if this does not hold up, *e.g.* X_{chimp} does not contain SAVs, when compared to X_{human}; on the other hand, X_{mouse} and X_{fly} do. This figure confirms the main trend: inter-species variants are shifted to the left (less effect) with respect to the 1KG SAVs between healthy people and the shift is higher the more divergent an organism from human (e.g. human-chimp shifted less to the right than human-fly).

Fig. S4:

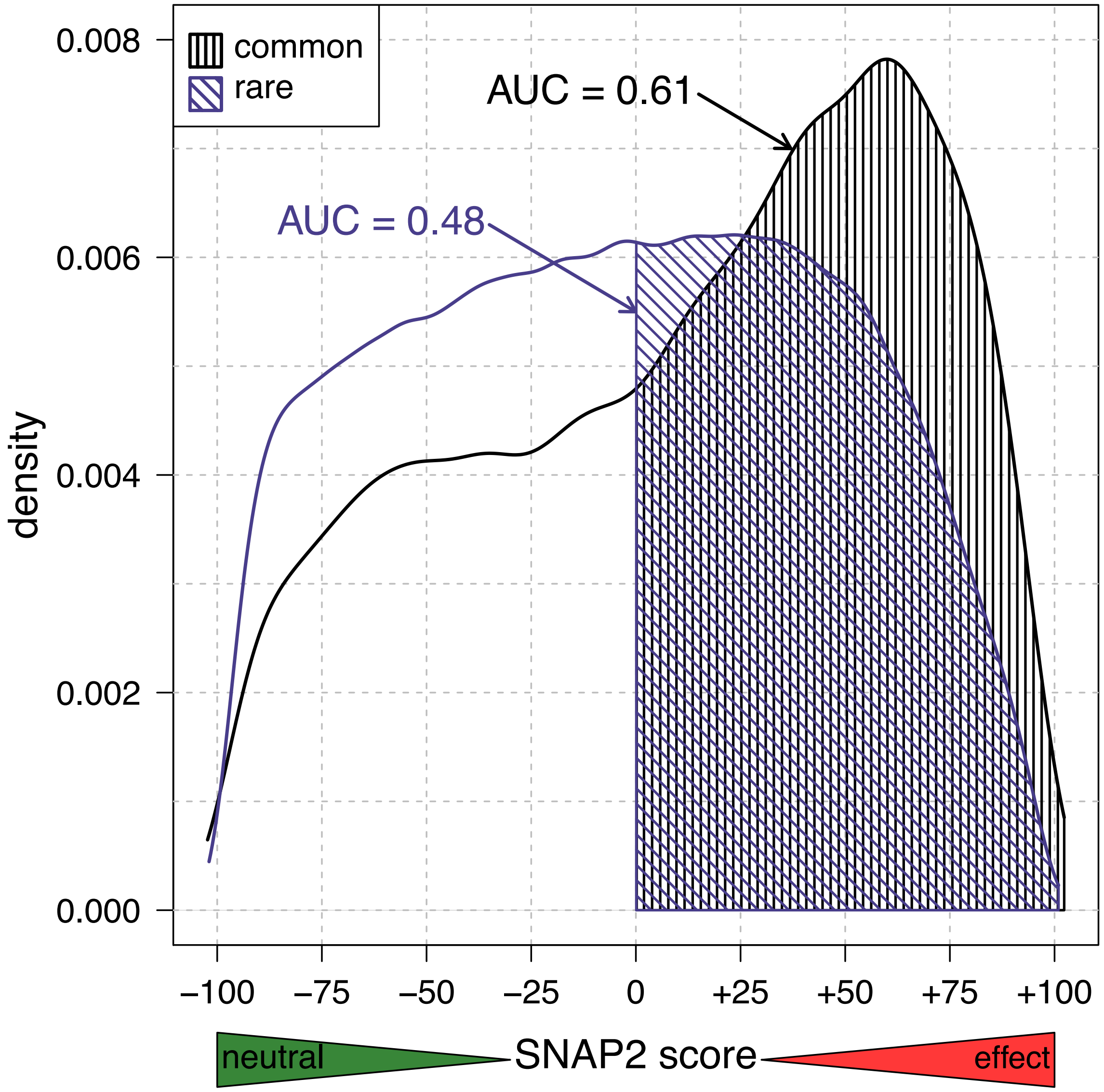

Fig. S4: SNAP2 predicts more common than rare SAVs to be effective. Displayed are the density curves of predicted SNAP2 scores for common (black) and rare (dark blue) 1KG SAVs. Examining the area under the curve for effect scores (SNAP score >= 0, common shaded black vertical, rare shaded dark blue diagonally) of both curves it is clearly visible that SNAP predicts a larger fraction of common SAVs to be effective than rare SAVs (AUC 0.61 vs. 0.48).

Fig. S5:

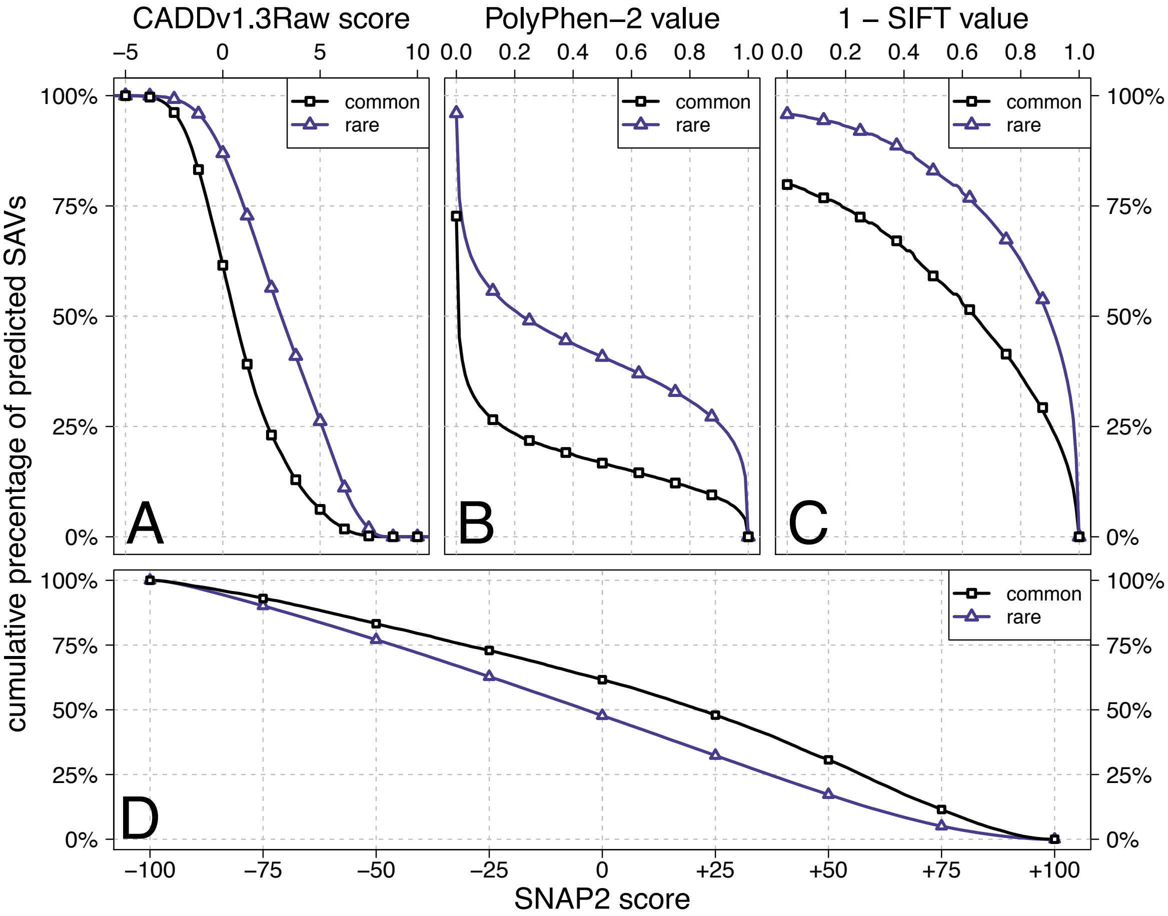

Fig. S5: CADD, PolyPhen-2 and SIFT predict a higher fraction of rare variants to be functionally effective than common variants. The cumulative percentages (read as Y% of SAVs are predicted to have higher score than X) for predicted SAVs across the range of scores for each method displayed a clear trend. CADD (A), PolyPhen-2 (B) and SIFT (C) predict rare variants (dark blue, triangles) to impact function of the protein more often than common variants (black, squares). This is in stark contrast to our predictions for common and rare SAVs by SNAP2 (D).

Fig. S6:

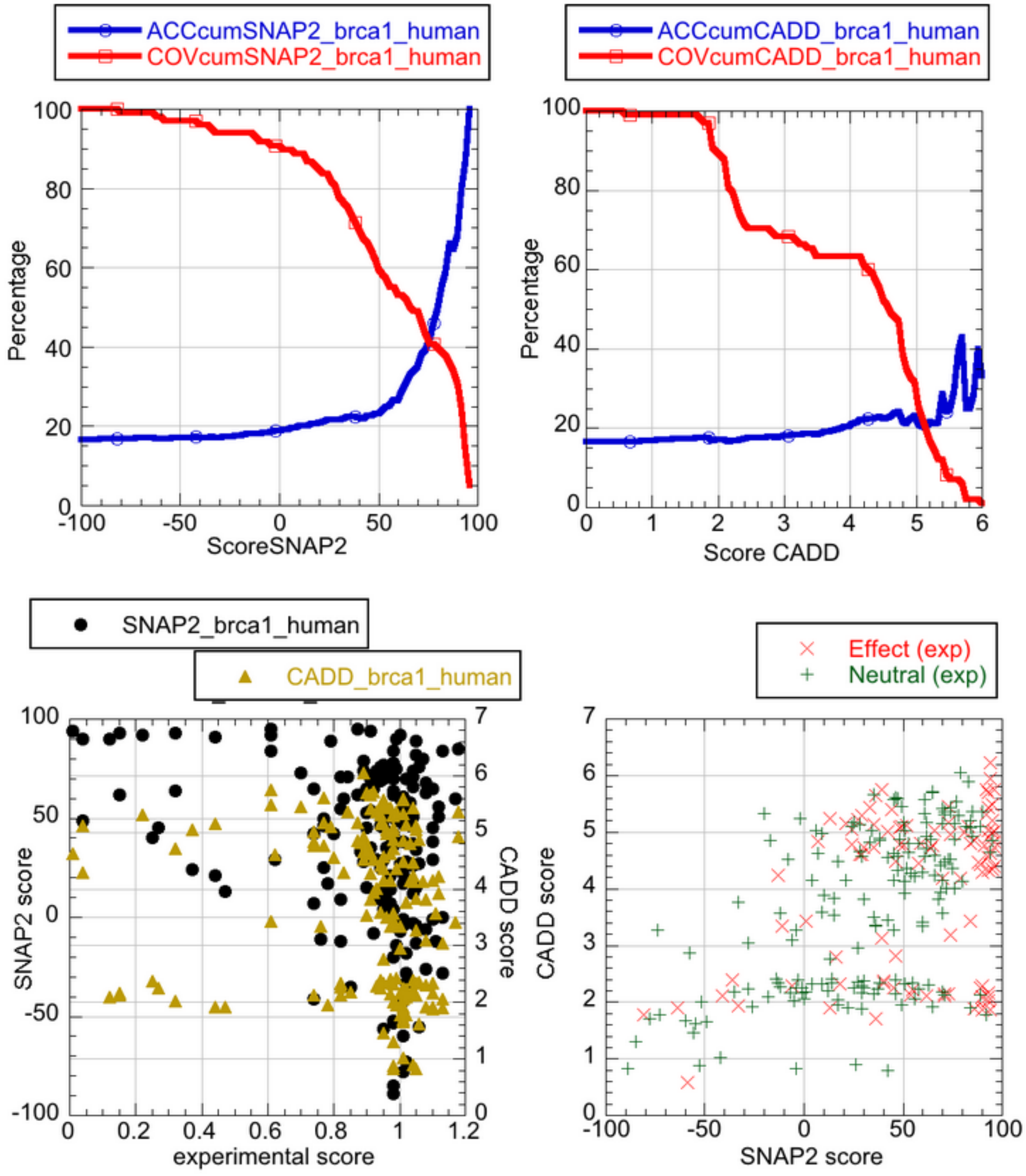

Fig. S6: SNAP2 captured molecular function better than CADD for deep scanning BRCA1 dataset. The lower left panel showed the experimental score from an experimental deep scanning testing the impact of SAVs upon molecular function (xxbr: put in quote of experiment here). SNAP2 results marked by black disks; the SNAP2-scores on the left y-axis ranged from strongly predicted as neutral (-100, bottom of the y-axis) to strongly predicted as effect (+100, top of the y-axis). The yellow-brown triangles gave results for CADD; CADD-scores on the right y-axis of the left panel. Visually, it seems that

SNAP2 correlated much better with the experimental score than CADD. The lower right panel tried to simplify by projecting the raw experimental score upon a binary classification, i.e. effect (red x) vs. neutral (green +). Using any threshold for SNAP2 and CADD scores (defaults: effect: SNAP2-score>0 and CADD>3), SNAP2 captured the simplified experimental impact of sequence variation upon molecular function better than CADD. The upper panels explicitly compile the performance for SNAP2 (upper left panel; x-axis SNAP2-score) and CADD (upper right panel; x-axis CADD-score). The y-axis of the upper panels showed cumulative percentages (blue line: accuracy=correctly predicted as effect/all predicted as effect; red line: coverage=correctly predicted as effect/all observed as effect). For instance, while for about 20% of the most strongly predicted SAVs SNAP2 reached over 80% accuracy (SNAP2-score>90: red curve ~20%, green curve ~80%), while CADD saturated at 20% accuracy for the same coverage (CADD-score>5: red curve ~20%, green curve ~20%).

Note that CADD never attempted to predict the impact of sequence variation upon molecular function. This figure gives one particular example proving that SNAP2 achieves the goal it was optimized for better than CADD the goal it was NOT optimized for. Although this was not much of a surprise, in light of the reverse of the predicted effect for rare and common SAVs, this confirmation constituted important evidence. The BRCA1 data was chosen for the only reason that it was the first large experimental deep scanning experiment that was made available to us. In a separate analysis, we have recently compiled additional data sets that confirm these findings for larger data sets (Theresa Wirth, TUM, in preparation); similar findings have been submitted by others (Thomas Hopf, Chris Sander & Debbie Marks, Harvard University)

Supplementary Note – SNAP2 training data

SNAP2 was trained on a set of ~100k mutations. The majority of these (~52%) were obtained from experimental effect annotations recorded in the Protein Mutant Database (PMD [2]). OMIM [3] and HumVar [4] effect variants accounted for another ~22% of the data and a set ~26% putative neutral variants was derived from alignments of enzymes with identical EC (Enzyme Commission) numbers [5]. Note that some of the latter might not be actually neutral due to compensating mutations (*i.e.* other sequence differences in same alignment), as well as due to differences in levels of ortholog activity between species. To avoid introducing a bias in this study towards predicting variants in orthologous sequences as neutral, we excluded all variants that were used for SNAP2 training from comparison.

References for Supporting Online Material

1. Bromberg, Y., Kahn, P.C. & Rost, B. Neutral and weakly nonneutral sequence variants may define individuality. *Proceedings of the National Academy of Sciences of the United States of America* **110**, 14255-14260 (2013).
2. Kawabata, T., Ota, M. & Nishikawa, K. The Protein Mutant Database. *Nucleic acids research* **27**, 355-357 (1999).
3. Hamosh, A., Scott, A.F., Amberger, J.S., Bocchini, C.A. & McKusick, V.A. Online Mendelian Inheritance in Man (OMIM), a knowledgebase of human genes and genetic disorders. *Nucleic Acids Res* **33**, D514-517 (2005).
4. Capriotti, E., Calabrese, R. & Casadio, R. Predicting the insurgence of human genetic diseases associated to single point protein mutations with support vector machines and evolutionary information. *Bioinformatics* **22**, 2729-2734 (2006).
5. Webb, E.C. Enzyme Nomenclature 1992. Recommendations of the Nomenclature committee of the International Union of Biochemistry and Molecular Biology., Edn. 1992. (Academic Press, New York; 1992).
